# Electrical Control of Uniformity in Quantum Dot Devices

**DOI:** 10.1021/acs.nanolett.2c04446

**Published:** 2023-03-28

**Authors:** Marcel Meyer, Corentin Déprez, Timo R. van Abswoude, Ilja N. Meijer, Dingshan Liu, Chien-An Wang, Saurabh Karwal, Stefan Oosterhout, Francesco Borsoi, Amir Sammak, Nico W. Hendrickx, Giordano Scappucci, Menno Veldhorst

**Affiliations:** † QuTech and Kavli Institute of Nanoscience, Delft University of Technology, PO Box 5046, 2600 GA Delft, The Netherlands; ‡ QuTech and Netherlands Organisation for Applied Scientific Research (TNO), PO Box 155, 2600 AD Delft, The Netherlands

**Keywords:** quantum dot, hysteresis, uniformity, spin qubit

## Abstract

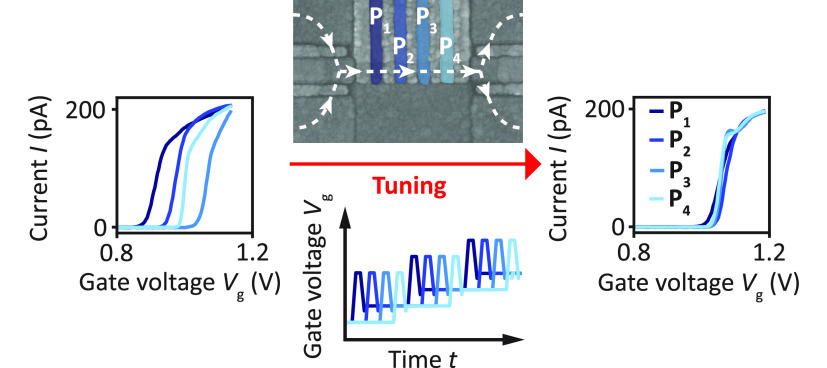

Highly uniform quantum systems are essential for the practical implementation of scalable quantum processors. While quantum dot spin qubits based on semiconductor technology are a promising platform for large-scale quantum computing, their small size makes them particularly sensitive to their local environment. Here, we present a method to electrically obtain a high degree of uniformity in the intrinsic potential landscape using hysteretic shifts of the gate voltage characteristics. We demonstrate the tuning of pinch-off voltages in quantum dot devices over hundreds of millivolts that then remain stable at least for hours. Applying our method, we homogenize the pinch-off voltages of the plunger gates in a linear array for four quantum dots, reducing the spread in pinch-off voltages by one order of magnitude. This work provides a new tool for the tuning of quantum dot devices and offers new perspectives for the implementation of scalable spin qubit arrays.

Spin qubits in semiconductor quantum dots are a promising platform for quantum information processing.^1−4^ Group IV semiconductors such as silicon and germanium can be isotopically purified,^5^ enabling long quantum coherence^6,7^ high-fidelity single-qubit^8−10^ and two-qubit gates^11−13^ as well as multi-qubit operation.^14,15^ Spin qubits can be operated at comparatively high temperatures,^16−18^ and their compatibility with semiconductor technologies spurred the realization of qubits made in industrial foundries.^19,20^ However, implementing more than a few qubits on a single chip remains extremely challenging.

Variations, in particular at the nanoscale, may lead to significant alterations of the relevant device metrics,^1,2,21^ such as the voltage needed to load a single electron to be used as a spin qubit. These variations can complicate the tuning of initialization, control, or readout and potentially form a roadblock for larger systems. Additionally, qubit-to-qubit variability may require the use of individual control electronics for each qubit, as is common practice in current experimental implementations, thus challenging the scalability. While several proposals have been put forward to scale quantum dot qubits,^2,22−24^ in all cases a high level of device uniformity is critical in their realization.

For semiconductor quantum dot qubits, the uniformity of the potential landscape is the key parameter that dictates the number of control voltages required per qubit. Ideally, a few voltages would suffice to induce a highly regular potential landscape as, drawn in Figure 1b. Yet, potential fluctuations are naturally present, as illustrated in Figure 1c. They can be caused by defects, charge traps and mechanical stress induced by the deposition of metallic gates,^25,26^ as well as variations in material growth or in the exact shape of the gates. The development of devices based on quantum wells buried in heterostructures, similar to that sketched in Figure 1a, already has led to a drastic improvement of the uniformity compared to metal oxide semiconductor systems.^27^ This has enabled the control of up to 16 quantum dots in a 4 × 4 array with shared gate control.^28^ However, significant variations in the quantum dot potential landscape are still commonly observed.^28−30^ This raises the question whether material^4^ and fabrication development^20,28,31,32^ will suffice to reach the required uniformity to operate large qubit arrays.

**Figure 1:**
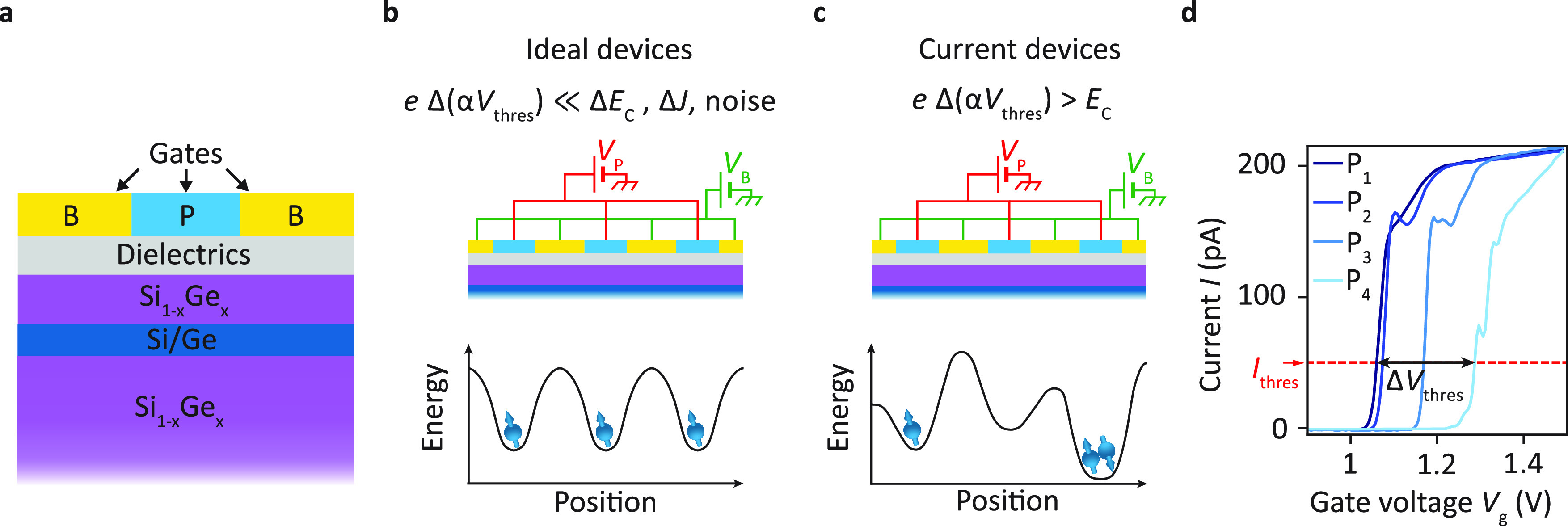
Fluctuations in the potential landscape in semiconductor quantum dot devices. (a) Schematic of typical semiconductor heterostructures with buried quantum wells studied. The metallic gate electrodes colored in yellow and blue represent the barrier (B) and plunger gates (P) of a quantum dot array, respectively. (b) Potential landscape in an ideal device with shared gate control. The application of the same voltage *V*_P/B_ on all plunger/barrier gates leads to a regular potential landscape with negligible fluctuations compared to those of the other relevant energy scales (α denotes the gate lever arm). The quantum dots all have the same charge configuration. (c) Potential landscape in state-of-the-art devices with shared gate control. The application of the same voltage *V*_P/B_ on all plunger/barrier gates leads to an irregular potential landscape due to local fluctuations, which are often comparable to or larger than the charging energy *E*_C_. Consequently, the quantum dots have different charge configurations. (d) Typical variations in the pinch-off characteristic of the plunger gates in a state-of-the-art linear quantum dot array (device A), nominally identical with that displayed in in Figure 4a, just after a cooldown. The pinch-off voltage *V*_thres_ is defined as the gate voltage for which the current reaches *I*_thres_ = 50 pA at a bias of |*V*_sd_| = 100 μV. Here, the pinch-off voltages spread over a voltage range Δ*V*_thres_ = 226 mV.

Here, we present an alternative method and demonstrate electrical control of uniformity in quantum dot devices. Our approach takes advantage of the gate voltage hysteresis, a ubiquitous effect observed in semiconductor heterostructures, that is mostly considered as a limitation in the tune-up of quantum dots. It manifests in shifts of the gate voltage characteristics and is commonly explained by a buildup of charges at the interface between the semiconductor barrier and the dielectrics, which then alter the electric field in the buried quantum well.^33−39^ We unveil the hysteresis and its effects on the potential landscape beneath the gates by studying how pinch-off characteristics evolve with the application of tailored stress voltage sequences. This method allows us to tune those pinch-off voltages over hundreds of millivolts, after which they remain stable at least on the time scale of hours. We then apply our findings to homogeneize the plunger gate pinch-off characteristics in a linear quantum dot array, reducing potential fluctuations in the quantum well underneath the corresponding gates.

The gate voltage required to confine a single electron or hole typically varies between quantum dots in an array, as it is dependent on the local electrostatic environment. These fluctuations also affect the pinch-off curve as exemplarily depicted for sweeping the four plunger gates of a linear quantum dot device (similar to that shown in Figure 4a) in Figure 1d. The curves reveal the local depletion of a conducting path through the quantum well and experimentally can be obtained in a very short time compared to the time required for the formation of a well-defined quantum dot. Therefore, we will employ pinch-off characteristics in the following to efficiently estimate variations in the potential landscape on the length scale of single quantum dots. In particular, we focus on the pinch-off voltages *V*_thres_ defined as the gate voltages at which a current of *I*_thres_ = 50 pA is reached for an applied source drain bias of |*V*_sd_| = 100 μV.

We study devices in ^28^Si/SiGe heterostructures^40^ and investigate how the pinch-off voltage of a single gate evolves depending on the previously applied gate voltages. To that end, we conduct systematic transport measurements at 4.2 K similar to sequences in refs (41−44) following the procedure depicted in Figure 2a. First a stress voltage *V*_stress_ is applied to the gate under study for a time *t*_stress_ = 1 min. Then the gate voltage is swept back until the pinch-off condition *I* = *I*_thres_ is met. This sequence is repeated several times with evolving stress voltages to measure the evolution of *V*_thres_ as a function of *V*_stress_. First, the applied stress voltage *V*_stress_ is decreased stepwise to be increased gradually again after reaching a reversal point *V*_stress_ = *V*_stress_^rev^.

**Figure 2:**
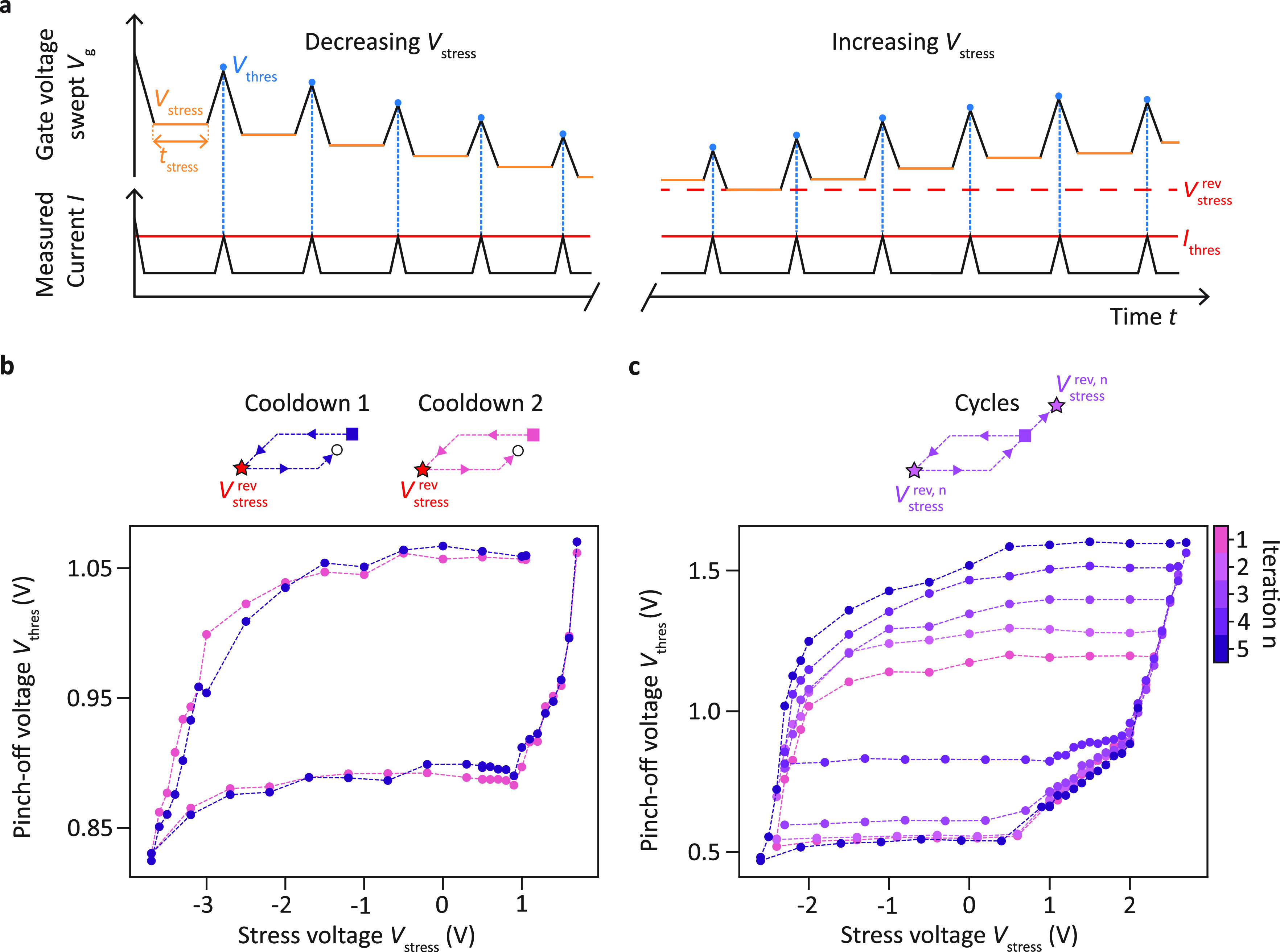
Hysteresis of the pinch-off characteristics. (a) Schematics of the measurement sequence used to probe the hysteretic behavior of the pinch-off voltage *V*_thres_ of a single gate. *V*_thres_, i.e. the voltage when the current reaches *I*_thres_ = 50 pA at a bias voltage |*V*_sd_| = 100 μV, is measured after application of successive stress voltages *V*_stress_ for *t*_stress_ = 1 min. The measurements start with decreasing *V*_stress_. Upon reaching *V*_stress_ = *V*_stress_^rev^, the direction is reversed and a sequence of increasing *V*_stress_ is applied. (b) Evolution of the pinch-off voltage *V*_thres_ of the sensor plunger gate S_1_ as a function of the stress voltage *V*_stress_ for two different cooldowns of device B. The measurement cycle is sketched in the top illustration. The square and the circle mark the starting point and the ending point of the cycles, respectively. The star indicates the point *V*_stress_^rev^ where the stress voltage sequence is reversed. *V*_stress_ is first decreased before being increased again after *V*_stress_^rev^ = −3.7 V. Both sets of points draw hysteresis cycles which overlap. The remaining gates that are needed to form a conductive channel are set to *V*_0_ = 1.2 V. (c) (*V*_stress_, *V*_thres_) hysteresis cycles measured successively for plunger gate P_1_ in device A. The points where the stress voltage sequences are reversed (stars) and ended (circles) are changed between each cycle. Note that for the fifth iteration, the stress voltage sequence with increasing *V*_stress_ was stopped purposely when *V*_thres_ ≃ 1 V. All other gates were set to *V*_0_ = 1.704 V.

Figure 2b shows the resulting pinch-off voltage evolution for a plunger gate P_*i*_ that is part of a linear quantum dot array for two different cooldowns (blue and pink curve, respectively). In these cases, *V*_stress_ is first lowered stepwise from *V*_stress_ = 1.05 V to *V*_stress_^rev^ = −3.7 V. We observe that up to *V*_stress_ > −2.0 V the pinch-off voltages *V*_thres_ stay within ±15 mV of the first pinch-off voltage *V*_thres_^0^ = 1.06 V, forming a plateau. Then, they drop down rapidly to *V*_thres_ = 0.83 V. At *V*_stress_^rev^ = −3.7 V, the sweep direction is reversed and we start to increase *V*_stress_ progressively. However, we do not observe a reversed behavior. Instead, from *V*_stress_ = −2.7 V to *V*_stress_ = 0.9 V, the pinch-off voltages increase by less than 25 mV, forming a second plateau. Only when *V*_stress_ = 1.0 (1.1) V for the first (second) cooldown does *V*_thres_ start to increase steeply again. The ensembles of (*V*_stress_, *V*_thres_) values draw typical hysteresis cycles with plateaus marking the ranges of applicable gate voltages over which the pinch-off voltage is not significantly changing. Furthermore, Figure 2b highlights the effect of thermal cycling on these measurements and reveals a remarkable overlap of the hysteresis cycles measured during two different cooldowns. A high degree of similarity is also observed when comparing successive measurements performed using the same stress voltage sequence as shown in Figure S2 in the Supporting Information for gate S of device D. This suggests that the underlying process has a deterministic nature.

Similar experiments performed on another sample with varying reversal points *V*_stress_^rev^ result in the cycles plotted in Figure 2c. The shapes of the curves are nearly identical for each iteration. Again, we observe plateaus where the pinch-off voltage deviates by less than 50 mV from its first value. Yet, the position of the plateaus varies with the chosen *V*_stress_^rev^. The pinch-off voltage plateaus can be shifted by up to |Δ*V*_thres_| = 290 mV for the lower plateau and by up to |Δ*V*_thres_| = 400 mV for the upper plateau. Overall, Figure 2b,c suggests that by applying a dedicated voltage sequence the pinch-off voltage can be adjusted on demand to chosen targets and thus that the intrinsic potential landscape underneath the gates can be tuned.

We also note that similar hysteretic behaviors, with sample-dependent variations of the exact shape of the (*V*_stress_, *V*_thres_) curves, are consistently found in several Si/SiGe devices (e.g., device D gate S shown in Figure S2 in the Supporting Information) as well as in samples made from Ge/SiGe heterostructures (see Figure S3 in the Supporting Information), suggesting a common underlying mechanism. The observed reproducibility and the large control window of the pinch-off voltage are the foundations of our approach to homogenize the potential landscape below an ensemble of gates.

However, the electrical tuning of the intrinsic potential uniformity is of practical interest only if the resulting potential landscape remains stable afterward. Therefore, we study how the pinch-off voltage evolves in time after stopping the hysteresis measurement cycle at varying points. The procedure followed is depicted in Figure 3a. The gate voltage is swept back and forth continuously to determine the voltage range  over which the current stays in a small range [*I*_thres_ – Δ*I*, *I*_thres_ + Δ*I*] around the current threshold *I*_thres_ as a function of time *t*. For each sweep  a linear regression *I* = *m* × *V* + *b* with fitting parameters *m* and *b* is applied from which the pinch-off voltage *V*_thres_(*t*) = (*I*_thres_ – *b*)/*m* is extracted. Figure 3a shows the time evolution directly after the application of decreasing (violet and blue) and increasing (light pink) stress voltages. For comparison, we also plot how the pinch-off voltage evolves right after a cooldown without prior application of a stress voltage sequence (dark pink). For decreasing *V*_stress_ sequences, the pinch-off voltages converge into steady states after initial decays and the time evolution exhibits random abrupt jumps. For the situation where no stress voltage or increasing stress voltages *V*_stress_ are applied, no significant variations of *V*_thres_ are observed. The relative evolution depicted in Figure 3b reveals that, for *t* > 2 h, the voltage fluctuations are similar for all three situations. This is confirmed by extracting the standard deviations of *V*_thres_ for experiments with and without application of stress voltage sequences, which are σ_stress_ = 0.4 mV (increasing *V*_stress_), σ_stress_ = 1.0 and 0.6 mV (decreasing *V*_stress_), and σ_no stress_ = 0.8 mV (no stress), respectively. These experiments suggest that after a potential initial transient regime there is no change in the stability of the device due to the electrical tuning. This stability is observed for at least 1 h and up to 3 h depending on the voltage sequence applied.

**Figure 3:**
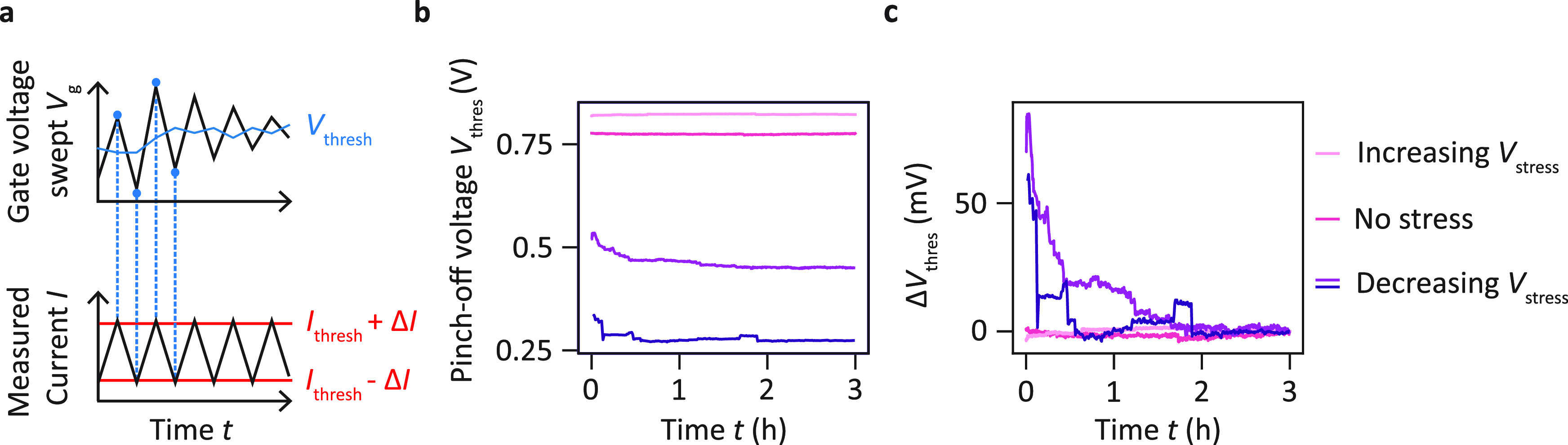
Stability of the pinch-off voltage after tuning. (a) Schematic representation of the procedure used to probe the time stability of the pinch-off voltage of a single gate. The gate voltage is continuously swept back and forth to detect the voltage range  over which the current stays between *I*_thres_ – Δ*I* and *I*_thres_ + Δ*I* with Δ*I* ∈ {10 pA, 25 pA}. From each sweep *V*_thres_(*t*) is extracted by linear regression. (b) Time evolution of *V*_thres_ prior to any application of stress voltages (dark pink) and after tuning via application of increasing *V*_stress_ with *V*_stress_^rev^ > 0 V (light pink) or decreasing *V*_stress_ with *V*_stress_^rev^ < 0 V (violet and blue). The curves are obtained for sensor plunger gate S in device C, except for the pink curve which is obtained for sensor plunger gate S in device D. *I*_thres_ = 50 pA, except for the blue curve of decreasing stress, where it was defined as *I*_thres_ = 30 pA, which provided a more robust analysis. (c) Relative variations Δ*V*_thres_(*t*) = *V*_thres_(*t*) – *V*_thres_(*t* = 3 h) of the data shown in (b).

Next we apply our findings and probe the capability to homogenize the pinch-off voltages *V*_thres_^i^ of a group of plunger gates P_*i*_ with *i* in [1,4] in a quantum dot array. Figure 4a displays the device studied, which has a geometry similar to linear quantum dot arrays in refs (12, 15, 29, and 45). The pinch-off characteristics recorded prior to the tuning sequence are depicted in the left panel of Figure 4c and show a spread Δ*V*_thres_ = max(*V*_thres_) – min(*V*_thres_) of 153 mV. Employing increasing gate voltage stress, we tune the individual plunger pinch-off voltages to the target value *V*_target_ = 1.05 V chosen before starting the tuning. Figure 4b illustrates the procedure followed for the specific case of two gates. A schematic representation including all four gates is displayed in Figure S4 in the Supporting Information. *V*_stress_ is gradually increased in *n* steps. For each *V*_stress_^*n*^, the plunger gates are sequentially stressed, measured, and parked Δ*V*_park_ = 50 mV above their latest pinch-off voltage, where they remain until the next stress voltage *V*_stress_^*n*+1^ = *V*_stress_^*n*^ + Δ*V*_stress_ is selected. When a pinch-off voltage *V*_thres_^i^ crosses the target voltage *V*_target_, the corresponding plunger gate P_*i*_ is henceforth no longer stressed. A full automated round of this sequence finishes after all pinch-off voltages are larger than the target voltage. The complete procedure is repeated two times with a stress voltage resolution of Δ*V*_stress_ = 25 mV taking approximately 9 h in total. All applied stress voltages and measured pinch-off voltages are visualized in the panels of Figure 4d. After each repetition a pinch-off characterization is performed with the resulting curves depicted in Figure 4c. During the first round the pinch-off voltages shift toward the target voltage *V*_target_ (indicated by the red dashed line), finally spreading in a range of Δ*V*_thres_ = 86 mV around it. This spread is further reduced by the following iteration, reaching a final value of Δ*V*_thres_ = 20 mV. Afterward the plunger pinch-off characteristics are observed to remain stable at least for 20 min (see Figure S5 in the Supporting Information).

**Figure 4:**
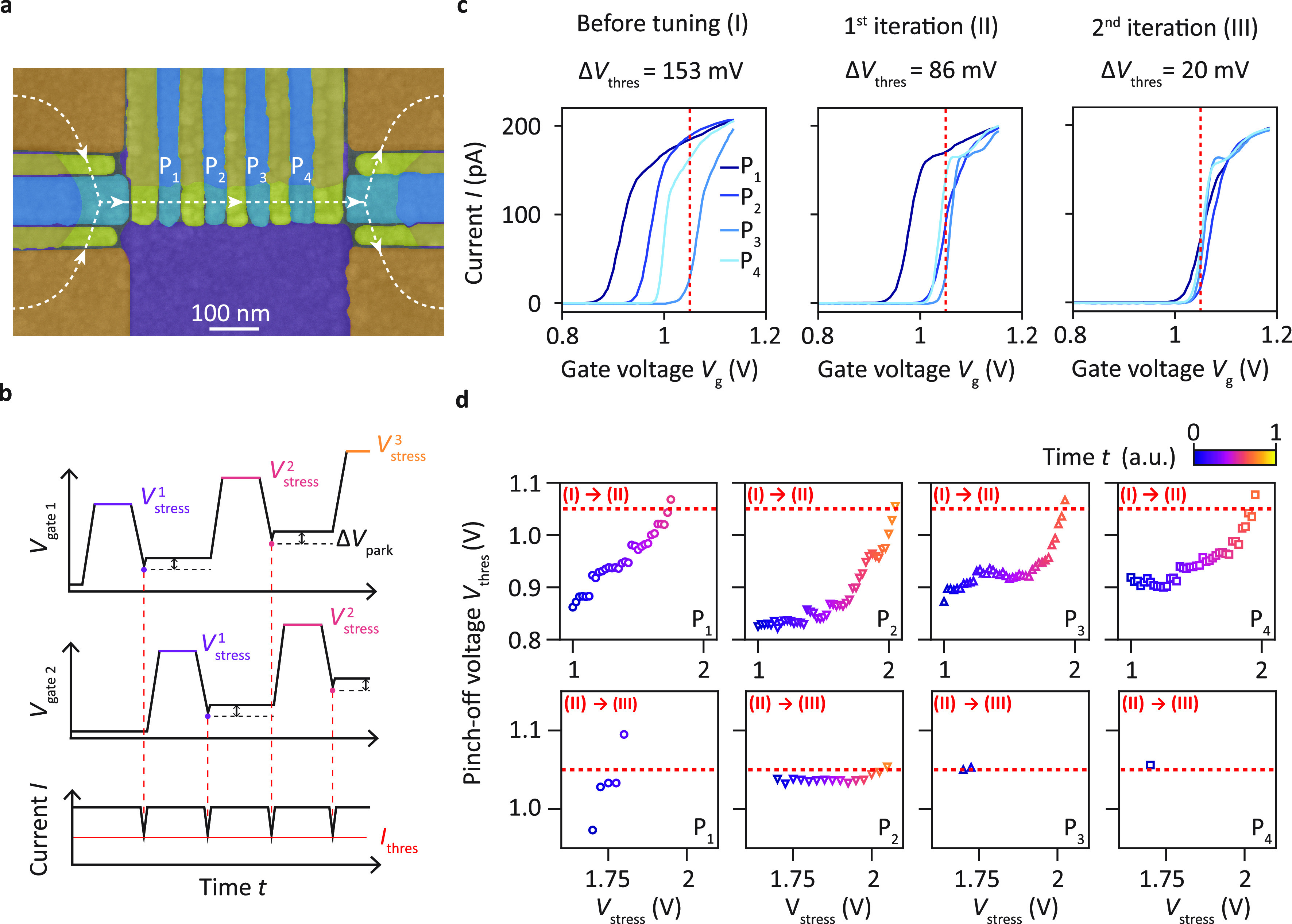
Homogenization of the potential landscape below the plunger gates of a linear quantum dot array. (a) Scanning electron micrograph of a linear quantum dot array. The plunger, barrier, accumulation, and screening gates are colored in blue, yellow, orange, and violet, respectively. The current flow is depicted by the dashed line. We aim at equalizing the pinch-off voltages of the plunger gates P_*i*_. (b) Schematics of the strategy followed illustrated with only two gates for clarity. Note that here, in contrast to the illustration in Figure 2a, the pinch-off voltage *V*_thres_ is detected through lowering the gate voltage until *I* = *I*_thres_. (c) Evolution of the pinch-off characteristics in device A after two iterations of the tuning procedure. The target voltage *V*_target_ = 1.05 V is marked by a red dashed line. After two iterations the spread of the pinch-off voltage Δ*V*_thres_ is reduced from 153 mV to 20 mV. (d) Evolution of *V*_thres_ for each gate while *V*_stress_ is increased during the tuning procedure. The red dashed line indicates the target pinch-off voltage *V*_target_ = 1.05 V. The stressing on each gate is stopped when its pinch-off voltage becomes larger than *V*_target_. The coloring of the data points encodes the time evolution of the stress and pinch-off voltages of the gates during each iteration.

To discuss our results and their implications for the tuning of quantum dot arrays, we assume that pinch-off voltages constitute a witness of the intrinsic potential landscape in the quantum well. Thus, we state that the observed tunability of pinch-off voltages also directly translates into a similar tunability of quantum dot chemical potentials. This statement is supported by a study of the effect of stress voltages on charge transitions of a quantum dot discussed in section VII in the Supporting Information. We find that the quantum dot potential can be tuned analogously to the threshold voltage *V*_thres_ by applying stress voltage sequences on the quantum dot plunger gate.

This motivates us to compare the final spread of the pinch-off voltages to the degree of uniformity needed to load an array of quantum dots with a single electron at each site using a single common gate voltage. Reaching such uniformity would require the potential fluctuations below the gates to be smaller than the average charging voltage *V*_C_ = *E*_C_/α that is needed to alter the charge occupation, with *E*_C_ being the charging energy and α the gate lever arm. Charging voltages typically range from 10 to 60 mV in devices similar to that under study.^27,29,45,46^ Consequently, the final spread Δ*V*_thres_ = 20 mV reached after electrical tuning promises a path toward the homogenization of quantum dot potentials inside an array. Even smaller spreads might be achievable by decreasing the stress voltage resolution Δ*V*_stress_. We envision that a similar method could be used to tune the potential underneath all plunger and all barrier gates simultaneously. It could also allow equalizing the interdot tunnel couplings and reaching an energy landscape similar to that in Figure 1b.

At the same time, optimization of the automated procedure could lead to a significant increase of the tuning efficiency. Such an optimized procedure may be obtained by dividing the tuning into coarse and fine steps and exploring different stressing times and thereby could drastically reduce the tuning time. Additionally, utilizing a model to predict the effect of the next stress voltage could further minimize the number of steps required to reach the target potentials, and simultaneous tuning of multiple gates may be envisioned in larger quantum dot arrays.

Adapted tuning procedures may also be designed for scalable device architectures. In a crossbar gate architecture,^24,28^ one could envision applying different stressing voltages on different sets of gates such that only close to the crossing points of these gates the combined electric field would be strong enough to shift the intrinsic potential. This would allow parallel but individual stressing of selected sites in a row-by-row manner. Another degree of selectivity might be provided through biasing of purposely isolated parts of the quantum well. Effectively, this would locally change the gates’ reference potential and thereby locally alter the effect of the stressing voltages applied to them. Further work is needed to confirm the viability of these approaches.

Also, a better understanding of the underlying mechanism of the hysteresis would be valuable to exploit it most efficiently. A possible origin might be the trapping and detrapping of charge in or close to the dielectric capping layer caused by the application of stress voltages.^33−38^ For example, a positive stress voltage might enable the tunneling of electrons from the quantum well or traps underneath nonstressed gates to traps underneath the stressed gate. These traps could be bound states in the nonoxidized part of the silicon capping layer or at its SiGe interface. They can be induced by charge defects in the gate oxide^47^ or emerge due to mechanical stress originating from the deposition of metallic gates.^25,26^ Also, charge trapping into and out of unpassivated silicon and germanium dangling bonds,^48−50^ charge trapping in the oxide itself mediated by leakage currents,^44,51−53^ or movement of mobile ions^54^ might be underlying the hysteresis. In all cases, when the gate voltage stress is removed, the charges would be expected to be immobile at the device operation temperature and would cause local shifts in the intrinsic potential landscape observable as alterations in the pinch-off characteristics. This tunneling and trapping of charge also would be highly similar to the principle used to operate modern flash memories (based on electrically erasable programmable read-only memories), which encode their stored information in pinch-off voltages and rely on gate stacks specifically engineered for that purpose.^53,55^ They could inspire new heterostructures and gate stacks with dedicated trapping layers, further refining the tunability of the potential landscape using the gate voltage hysteresis.

In conclusion, we have presented a new method to increase the electrostatic potential uniformity in quantum dot devices electrically. We demonstrate that we can take advantage of hysteretic shifts in gate voltage characteristics to deliberately tune pinch-off voltages across a wide range of more than 500 mV by applying stress voltage sequences. The resulting states remain stable on the time scale of hours. We also show that the chemical potential of single quantum dots can be tuned using similar procedures. Utilizing our method, we have shifted and equalized the pinch-off voltages of four plunger gates in a linear quantum dot array to a predetermined target voltage. Although most of our results were obtained in Si/SiGe heterostructures, other measurements indicate that the effect and method also can be used in other heterostructure materials like Ge/SiGe. Our work opens up a new path to increase uniformity in quantum dot based spin qubits. It may enable reducing overheads in tuning and control, making the implementation of scalable architectures more feasible in practice.

## Data Availability

The data and analysis supporting this work are openly available in a public Zenodo repository at 10.5281/zenodo.7746206.^56^

## Supporting Information Available

Materials and device fabrication details, description of the experimental setup, details on the experimental procedures, SEM images of devices nominally identical with those used in this work, presentation of ten consecutive hysteresis cycles, hysteresis cycles obtained in Ge/SiGe single hole transistor structures, full schematics of the stress voltage sequence employed for tuning the plunger gate pinch-off voltages in a linear array, stability of the four plunger pinch-off characteristics after the tuning procedure presented, analysis of the effect of stress voltages on coulomb-blockade oscillations, table of samples investigated and corresponding reversal points (PDF)
