# Supplementary material for: Electrical Control of Uniformity in Quantum Dot Devices
Source: Nano Lett. 2023 Mar 28;23(7):2522–9. doi: 10.1021/acs.nanolett.2c04446 (PMC10103318; doi:10.1021/acs.nanolett.2c04446)
Supplement: Supplementary file 1 — nl2c04446_si_001.pdf [file nl2c04446_si_001.pdf]

Supplementary Information: Electrical Control of Uniformity in Quantum Dot Devices

Marcel Meyer,[1] Corentin Déprez,[1] Timo R. van Abswoude,[1] Ilja N. Meijer,[1] Dingshan Liu,[1] Chien-An Wang,[1] Saurabh Karwal,[2] Stefan Oosterhout,[2] Francesco Borsoi,[1] Amir Sammak,[2] Nico W. Hendrickx,[1] Giordano Scappucci,[1] and Menno Veldhorst[1]

[1] *QuTech and Kavli Institute of Nanoscience, Delft University of Technology, PO Box 5046, 2600 GA Delft, The Netherlands*

[2] *QuTech and Netherlands Organisation for Applied Scientific Research (TNO), PO Box 155, 2600 AD Delft, The Netherlands*

(Dated: March 27, 2023)

I. MATERIAL AND METHODS

A. Materials and device fabrication

The devices studied here are made from ^{28}Si/SiGe heterostructures [1]. They are grown on top of a natural Si wafer and begin by a linearly graded $Si_{1-x}Ge_x$ wafer with x varying from 0 to 0.3. A relaxed $Si_{0.7}Ge_{0.3}$ layer of 300 nm is then grown, followed by a 9 nm purified ^{28}Si layer (with 800 ppm purity) and another 30 nm thick relaxed $Si_{0.7}Ge_{0.3}$ layer. Finally, an approximately $1-2$ nm thin Si capping layer is deposited. The 2DEGs are contacted via phosphorus ion implantation. Overlapping Ti/Pd gate electrodes are deposited via electron beam evaporation. The different sets of gates are separated from each other and from the Si capping layer by 5 nm and 10 nm aluminum oxide layers, respectively, deposited through atomic layer deposition [2].

B. Experimental set-up

All the measurements presented are dc-transport measurement performed at 4.2 K by dipping the devices directly in liquid helium. The gate voltages are swept using 18 bit precision digital-to-analog converters having a ± 4 V range of applicable voltages. The current is measured via a current-to-voltage converter and a Keithley digital multimeter at an applied source-drain bias of $|V_{\rm sd}| = 100\ \mu$V. The data acquisition, the application of the stress voltages and the successive pinch-off voltage measurements were performed automatically using a home-made Python program.

C. Experimental procedures

Prior to any experiment, the group and individual pinch-off voltages $V_{\rm thres}$ of all gates forming a given conduction channel are measured. The group pinch-off voltage is measured by sweeping all gates corresponding to the channel under investigation simultaneously until a current of typically 200 to 300 pA is reached. The corresponding voltage V_0 is the voltage at which the gates not under study are parked most of the time. The individual pinch-off voltages are then measured by sweeping each gate voltage down and back up again starting from V_0. Such measurements allow to characterize the potential uniformity just after the cooldown and to potentially discard malfunctioning devices. The measurement of individual pinch-off voltages is repeated between experiments to study how the spread of the pinch-off voltages evolves and thereby the potential uniformity. To that end, first all gate voltages responsible for forming the conducting path are set to the same value.

In most experiments, before recording a ($V_{\rm stress}$,$V_{\rm thres}$) curve, a minimum and a maximum threshold voltage $V_{\rm thres}^{\rm min}$ and $V_{\rm thres}^{\rm max}$ are defined. Once $V_{\rm thres} < V_{\rm thres}^{\rm min}$ or $V_{\rm thres} > V_{\rm thres}^{\rm max}$ the sequence of stress voltages $V_{\rm stress}$ is reverted defining a reversal point $V_{\rm stress}^{\rm rev}$. Furthermore, we define minimum and maximum stress voltages $V_{\rm stress}^{\rm min}$ and $V_{\rm stress}^{\rm max}$ for each cycle and if $V_{\rm stress} \leq V_{\rm stress}^{\rm min}$ or $V_{\rm stress} \geq V_{\rm stress}^{\rm max}$ the sequence is also reversed defining a reversal point $V_{\rm stress}^{\rm rev}$ as well. Tables summarizing the reversal points for the different experiments can be found in supplementary table T1 and supplementary table T2.

II. PRESENTATION OF THE SI/SIGE DEVICES INVESTIGATED

Here, we present the Si/SiGe quantum dot devices investigated in this work. Fig S1 shows typical scanning electron micrographs of the two types of devices. They are both composed of screening, accumulation/plunger and barrier

gates which are deposited in that order. The screening gates are usually kept close to 0 V to prevent the formation of conducting channels at undesired locations. The devices labeled A and B in the main text are nominally identical to the one presented in Fig S1.a. It is designed to form a linear array of four quantum dots with two larger quantum dots at the ends to be used as charge sensors. The devices named C and D are of the type displayed in Fig S1.b which shows a single quantum dot aimed to be a single electron transistor. It is located at the corner of a larger 3×3 quantum dot array (not shown here).

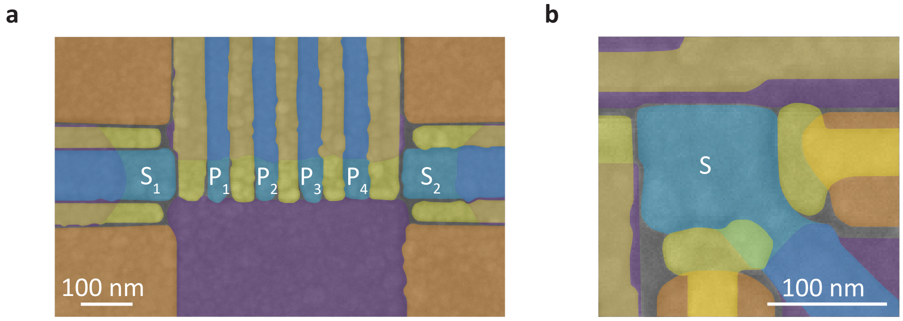

Figure S1. **Scanning electron micrograph of Si/SiGe devices studied.** **a**, Linear four quantum dot array, **b**, Single electron transistor at the edge of a 3×3 quantum dot array. The plunger gates are colored in blue, the barrier gates in yellow, the accumulation gates in orange and the screening gates in violet. In **a**, the plunger gates belonging to the linear channel are labelled P_i whereas that of the charge sensors are labelled S_i. In **b**, the plunger gate of the sensor used during the experiments is labelled S.

III. REPRODUCIBILITY OF THE (V_{thres},V_{stress}) HYSTERESIS CYCLES

To provide further evidence of the reproducibility of the hysteresis cycles, we perform an experiment where the same sequences of decreasing and then increasing stress voltages are repeated ten times for gate S in device D. To reduce the measurement time, we focus mostly on the voltage range where V_{thres} shows a strong evolution with V_{stress}. The results are displayed in Fig. S2.

We recognize the left and right flanks of the hysteresis cycles as well as the end of the V_{thres} plateaus for decreasing V_{stress}. Remarkably, the data obtained for the ten iterations collapse onto single curves both for increasing and decreasing V_{stress}. This further illustrates the high reproducibility of the (V_{thres},V_{stress}) hysteresis cycle that can be achieved.

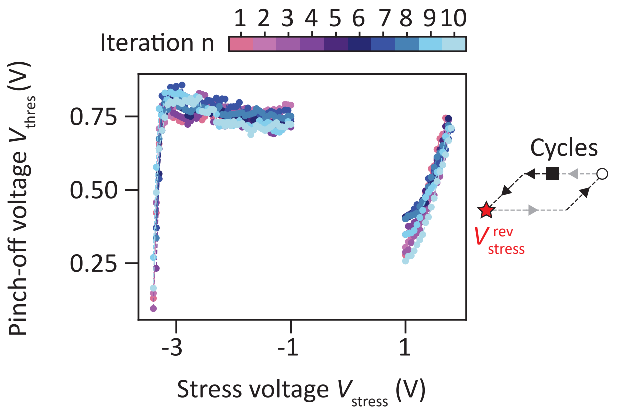

Figure S2. **Overlap of multiple hysteresis cycles.** Evolution of $V_{\rm thres}$ as a function of $V_{\rm stress}$ for 10 successive cycles obtained for gate S in device D. A schematic of the stress voltage sequence applied during one iteration is sketched on the right. The square and the circle mark the starting point and the ending point of the cycles, respectively. The star indicates the point where the stress voltage direction is reversed. The $V_{\rm thres}$ plateaus are not measured or only partially (grey dashed lines). All the curves collapse onto each other showing a remarkable reproducibility.

IV. PINCH-OFF VOLTAGES HYSTERESIS IN GE/SIGE

The hysteretic behavior of the pinch-off voltages and its dependence on the previous stress voltages applied is not exclusive to Si/SiGe heterostructures. The same effect can be observed in Ge/SiGe heterostructures. We perform experiments similar to those discussed in the main text in germanium single hole transistor (SHT) structures that are presented in Fig. S3.a. Note that these SHTs are part of a larger device presented in ref. [3].

The corresponding device is made from a strained Ge/SiGe heterostructures grown by chemical vapor deposition. Starting from a natural Si wafer, a 1.3 μm thick relaxed Ge layer is grown, followed by a 0.9 μm reverse graded $Si_{1-x}Ge_x$ (x going from 1 to 0.8) layer, a 500 nm relaxed $Si_{0.2}Ge_{0.8}$ layer, a 16 nm Ge quantum well under compressive stress, another 55 nm $Si_{0.2}Ge_{0.8}$ spacer layer and a $<$ 1 nm thick Si capping layer [4, 5]. The quantum well is contacted via 30-nm platinium contacts evaporated and diffused after etching of the oxidized Si capping layer [6]. Aluminum oxide layers of 7, 5, and 5 nm thickness grown by atomic layer deposition precede the deposition of overlapping Ti/Pd electrodes with thicknesses of 3/17, 3/27, 3/27 nm forming three different gate layers on top of the heterostructure [2].

We study the devices by applying a common gate voltage to the two barrier gates and the plunger gate defining the SHT such that a conductive channel is formed between the ohmic contacts. Fig. S3.b-d show typical hysteresis cycles obtained by measuring the evolution of the pinch-off voltage as function of the stress voltage applied on the three gates for each SHT. $V_{\rm stress}$ is first increased and then decreased in each measurement cycle contrary to the sequence followed in Fig. 2. These measurements are performed at base temperature of a dilution refrigerator and an estimated electron temperature of approximately 140 mK [3] and the pinch-off voltage was defined as the voltage at which the current reaches $I_{\rm thres} = 500$ pA at an source drain bias of $|V_{\rm sd}| = 100$ µV.

Overall, we observe similar features to those observed in the Si/SiGe devices i.e. overlapping hysteresis cycles with a tunable voltage range of a few hundred millivolts. These measurements highlight that the hysteresis of the pinch-off voltages is observable in multiple semiconductor heterostructures and that the tuning method presented in this work may be used in different materials as well.

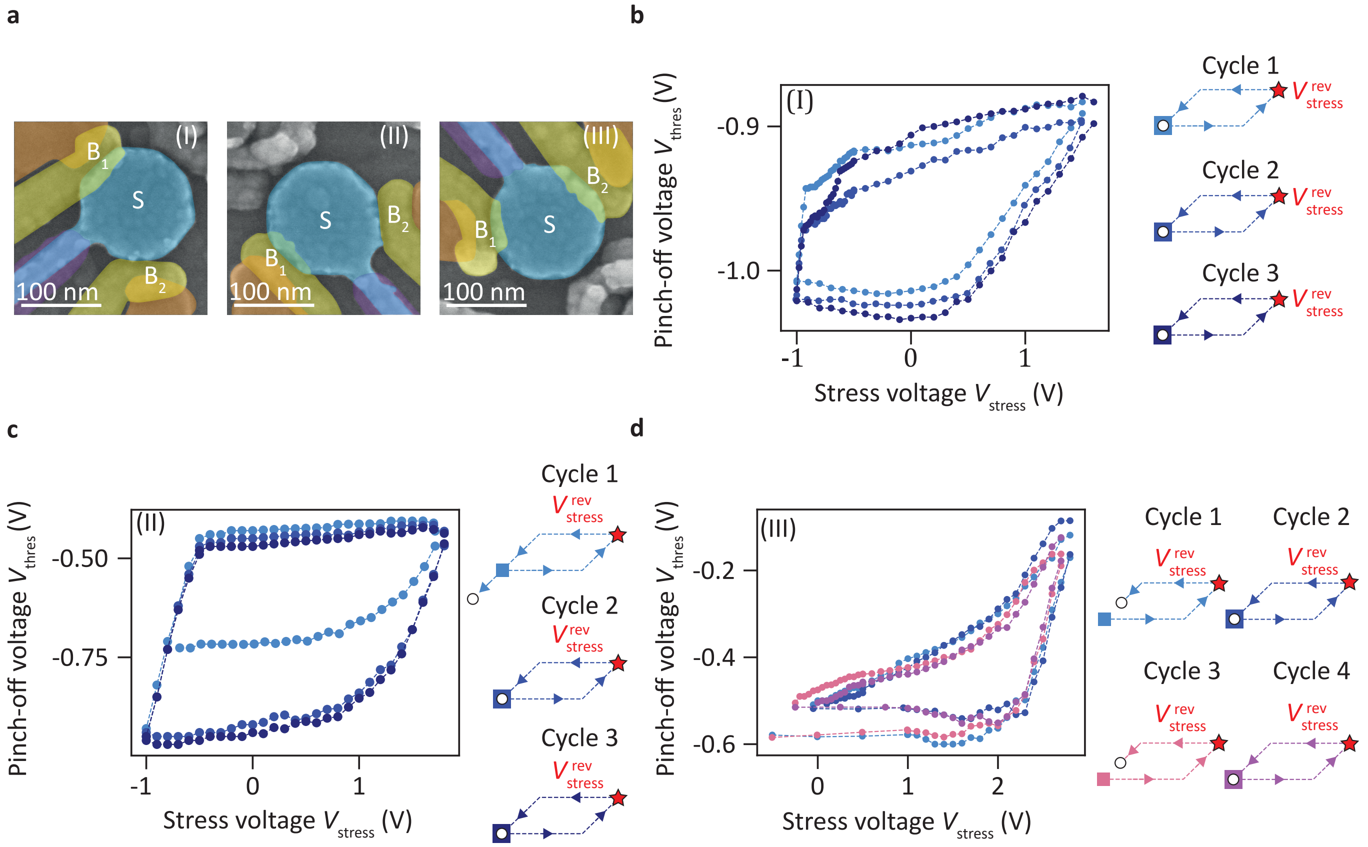

Figure S3. **Hysteresis of V_{thres} in Ge/SiGe single hole transistors.** **a**, Scanning electron micrograph of the SHTs studied. The plunger gates are colored in blue, the barrier gates in yellow, the ohmic contacts in orange and the screening gates in violet. The plunger gates of the SHTs used during the experiments are labelled S while the two barrier gates are labelled as $B_{1,2}$. **b**, **c**, **d**, Evolution of V_{thres} as a function of V_{stress} for successive stress voltage cycles applied simultaneously on the barrier gates and the plunger gate forming the SHT. The stress voltage cycles are depicted schematically on the right side. The square and the circle mark the starting point and the ending point of the cycles, respectively. The stress voltage $V_{\text{stress}}^{\text{rev}}$ upon which the stress voltage sequence is reversed is indicated by a star. For each SHT, the different data points overlap and form a hysteresis loop. In **d**, the device was kept idle for 5 hours between cycle 2 and cycle 3. This lead to a voltage difference $\Delta V_{\text{thres}} \approx -80$ mV between the last point of cycle 2 and the first point of cycle 3 similar to the time evolution discussed Fig. 3. The data is taken at an electron temperature of approximately 140 mK [3].

V. SEQUENCE OF STRESS VOLTAGE USED FOR THE TUNING OF THE PLUNGER GATES OF THE QUANTUM DOT 1D ARRAY.

We show a schematic of the stress voltage sequence applied on the plunger gates of the quantum dot 1D array to homogenize them and obtain the data displayed in Fig. 4. As discussed in the main text, the gates are sequentially stressed, one after the other, using the sequence of increasing stress voltages depicted in Fig. S4. After application of a stress voltage on a given gate, its pinch-off voltages is measured and then the voltage applied on it is increased by 50 mV. This voltage is kept constant during the stressing of the other gates and the characterization of their pinch-off voltage. Once the target threshold voltage is reached or exceed for one gate, we stop applying stress voltages to it.

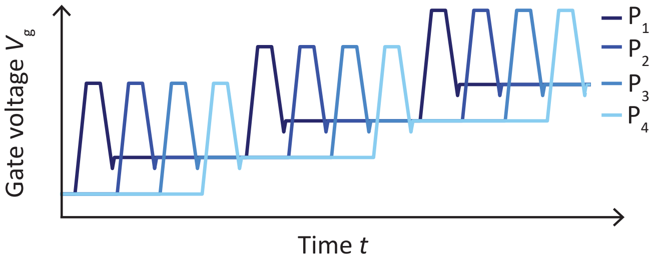

Figure S4. **Schematics of the voltage sequence used for the tuning of the plunger-gate pinch-off voltages.** A sequence of increasing stress voltages is applied to the plunger gates P_1-P_4 with each stress voltage being set sequentially. As soon as the pinch-off voltage of a plunger reaches the desired value the plunger is not stressed anymore (not shown).

VI. STABILITY OF THE PINCH-OFF CHARACTERISTICS AFTER TUNING THEM USING THE HYSTERETIC BEHAVIOUR

Here, we discuss the stability of the pinch-off voltages after reduction of their spread using the protocol presented in Fig. 4. Fig. S5 shows the pinch-off characteristics right after, 6, and 21 minutes after the tuning.

For each plunger gate considered, the three plots virtually overlap perfectly suggesting a high degree of stability. This also is in agreement with the absence of variations observed in the time stability measured after application of increasing stress voltages in Fig. 3. It supports our choice of using increasing stress voltages in the tuning procedure presented.

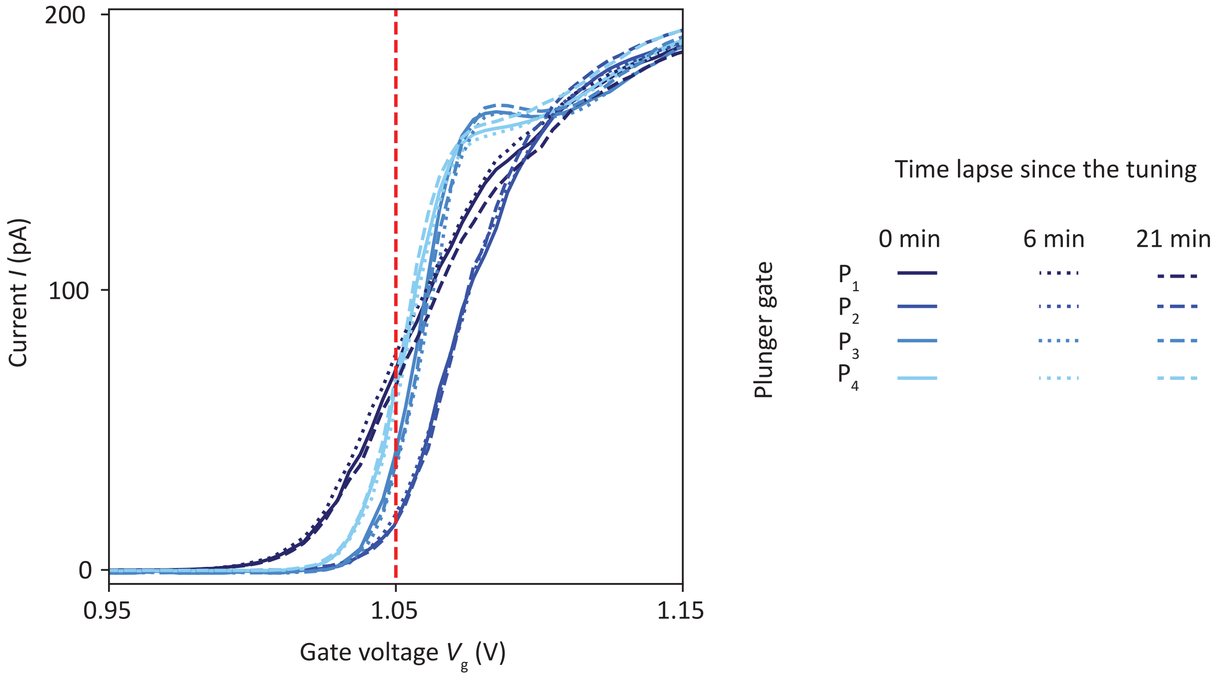

Figure S5. **Stability of the plunger gate pinch-off characteristics after tuning them to the target voltage.** Pinch-off characteristics measured just after (plain lines), 6 minutes (dotted lines) and 21 minutes (dashed lines) after the homogeneization procedure described in the main text using the hysteretic shift. The red dashed line marks the target voltage of the tuning.

VII. COULOMB-BLOCKADE HYSTERESIS

We also investigate the effect of applying stress voltages on a single quantum dot. Fig. S6.a shows a scanning electron micrograph of a silicon quantum dot device similar to the devices presented in the main text. The device is measured at 4.2 K and a source-drain voltage $|V_{\mathrm{sd}}| = 100$ µV is applied from left to right. This results in a current which flows as indicated by white dashed lines in Fig. S6.a. We first apply a full cycle of stress voltages to the plunger gate P_4, resulting in the (V_{stress},V_{thres}) hysteresis cycle depicted in Fig. S6.d which is very similar to that measured in other devices.

Next, we form a quantum dot under the plunger gate P_4 by tuning the voltages applied to P_4 and the surrounding barrier gates. The formation of the quantum dot is assessed by measuring the current flowing through the device as function of V_{sd} and the voltage applied on the plunger gate V_{P4}. Fig. S6.b shows the corresponding measurements. We observe Coulomb diamonds confirming the presence of a quantum dot underneath P_4. Similarly to measuring the V_{thres} hysteresis, we probe the evolution of Coulomb-blockade oscillations for a full cycle of stress voltages applied to P_4. Fig. S6.c contains a schematic representation of the measurement procedure. Here a full pinch-off curve is recorded after the application of each stress voltage. This procedure is repeated multiple times for sequences of increasing and decreasing stress voltages. The resulting pinch-off curves exhibit Coulomb-blockade oscillations, as illustrated in Fig. S7.a-d, from which we extract the voltages V_{t} where charge transitions occur using a peak detection routine. Fig. S6.e depicts the evolution of the first five visible charge transitions with the applied stress V_{stress}. Like in Fig. S6.d, a V_{t} hysteresis cycle is observed which we divide in four parts (labeled I to IV). Note that the amplitude of the detected peaks can vary along the cycle and that peaks might (dis)appear. Thus, the first five peaks might not always correspond to the same five charge transitions.

To identify the evolution of a specific charge transition $V_{\mathrm{t}}^{\mathrm{i}}$, we plot the corresponding Coulomb-blockade oscillations for successive stress voltages as shown in Fig. S7.a-d. For parts I and III, we observe that the V_{t} stay approximately constant from trace to trace shifting less than their spacing. This suggest that the chemical potential in the quantum dot and the confinement remain mostly unchanged. It also allows to identify the voltages $V_{\mathrm{t}}^{\mathrm{i}}$ corresponding to a specific charge transition for each stress voltage. For this purpose, we compare successive traces j and $j+1$ and we assign peak labels (coloured markers) that minimize the charge transition voltage shift $\Delta V_{\mathrm{t}}^{\mathrm{i}} = |V_{\mathrm{t}}^{\mathrm{i}}(V_{\mathrm{stress}}^{j}) - V_{\mathrm{t}}^{\mathrm{i}}(V_{\mathrm{stress}}^{j+1})|$. By minimizing $\Delta V_{\mathrm{t}}^{\mathrm{i}}$, we also find the labelling that minimizes changes of the corresponding peak heights $I_{\mathrm{peak}}^{\mathrm{i}} = I(V_{\mathrm{t}}^{\mathrm{i}})$ between two traces as depicted in Fig. S7.e and Fig. S7 g. It strengthens our confidence in the labelling of the charge transitions. We remark that in part I, around $V_{\mathrm{stress}} = -1.2$ mV, the amplitudes of Coulomb peaks all exhibit a sudden jump. As the peak height is an indicator of the tunnel coupling between the quantum dot and its leads, we interpret this jump as a sudden change of tunnel coupling. We see no evidence for a peak shift in the corresponding Coulomb-blockade oscillation traces. This suggest the application of stress voltages can also affect the tunnel coupling independently of the quantum dot potential.

In contrast, for parts II and IV, we observe significant shifts of the charge transitions from trace to trace. Therefore, in order to identify specific charge transitions, we look for minimal shifts $\Delta V_{\mathrm{t}}^{\mathrm{i}}$ but we also impose a monotonous evolution of the peak height $I_{\mathrm{peak}}^{\mathrm{i}}$ and minimize its changes $\Delta I_{\mathrm{peak}}^{\mathrm{i}} = |I_{\mathrm{peak}}^{\mathrm{i}}(V_{\mathrm{stress}}^{j}) - I_{\mathrm{peak}}^{\mathrm{i}}(V_{\mathrm{stress}}^{j+1})|$. Thus this labeling assumes that the dot tunnel coupling evolves continuously with the applied stress voltages. The assumption is strengthened by smooth $I_{\mathrm{peak}}^{\mathrm{i}}$ evolutions depicted in Fig. S7.f and Fig. S7.h. The imposed constrains result in a peak identification with, on part II, $V_{\mathrm{t}}^{\mathrm{i}}$ decreasing with decreasing stress voltages and, on part IV, $V_{\mathrm{t}}^{\mathrm{i}}$ increasing with increasing stress voltages. Thus, we recover the dependence observed in Fig. S6.d. As the charging voltages $V_{\mathrm{C}} = V_{\mathrm{t}}^{\mathrm{i}+1} - V_{\mathrm{t}}^{\mathrm{i}}$ plotted in Fig. S7.i-l remain unaffected, we attribute these shifts to changes in the quantum dot chemical potential and changes in the dot coupling to its leads.

While the identification of charge transition voltages $V_{\mathrm{t}}^{\mathrm{i}}$ (coloured markers in Fig. S6.e and Fig. S7) might not be unique for a few selected traces, overall we observe a clear trend in $V_{\mathrm{t}}^{\mathrm{i}}$. The quantum dot chemical potential follows an hysteresis cycle (Fig. S6.e) similar to the pinch-off voltages V_{thres} hysteresis (Fig. S6.d). To quantify this similarity, we extract the slopes $\mathrm{d}V_{\mathrm{thres}}/\mathrm{d}V_{\mathrm{stress}}$ and $\mathrm{d}V_{\mathrm{t}}^{\mathrm{i}}/\mathrm{d}V_{\mathrm{stress}}$ for parts II and IV by fitting the data with linear functions. For the V_{thres} hysteresis cycle, we fit only the points marked with diamonds in Fig. S6.d. For the $V_{\mathrm{t}}^{\mathrm{i}}$ hysteresis, we fit the charge transitions marked by dark blue circles. For part II, we extract $\mathrm{d}V_{\mathrm{thres}}/\mathrm{d}V_{\mathrm{stress}} = 2.77 \pm 0.35$ and $\mathrm{d}V_{\mathrm{t}}^{\mathrm{i}}/\mathrm{d}V_{\mathrm{stress}} = 3.6 \pm 0.25$. For part IV, we obtain $\mathrm{d}V_{\mathrm{thres}}/\mathrm{d}V_{\mathrm{stress}} = 0.51 \pm 0.01$ and $\mathrm{d}V_{\mathrm{t}}^{\mathrm{i}}/\mathrm{d}V_{\mathrm{stress}} = 0.87 \pm 0.06$. On both parts of the hysteresis cycles, the slopes obtained by fitting the $V_{\mathrm{stress}}/V_{\mathrm{thres}}$ and the $V_{\mathrm{stress}}/V_{\mathrm{t}}$ hysteresis cycles do not differ substantially confirming that the V_{thres} and the $V_{\mathrm{t}}^{\mathrm{i}}$ hysteresis cycles have a similar shape.

In summary, this set of measurements shows that the application of stress voltages allows to shift the chemical potential of a quantum dot. The observed hysteresis of the dot chemical potential is highly similar to that of the pinch-off voltage hysteresis for the same plunger gate. This suggests that pinch-off voltages and charge transition voltages can be equivalently utilized to witness changes in the intrinsic potential underneath a gate.

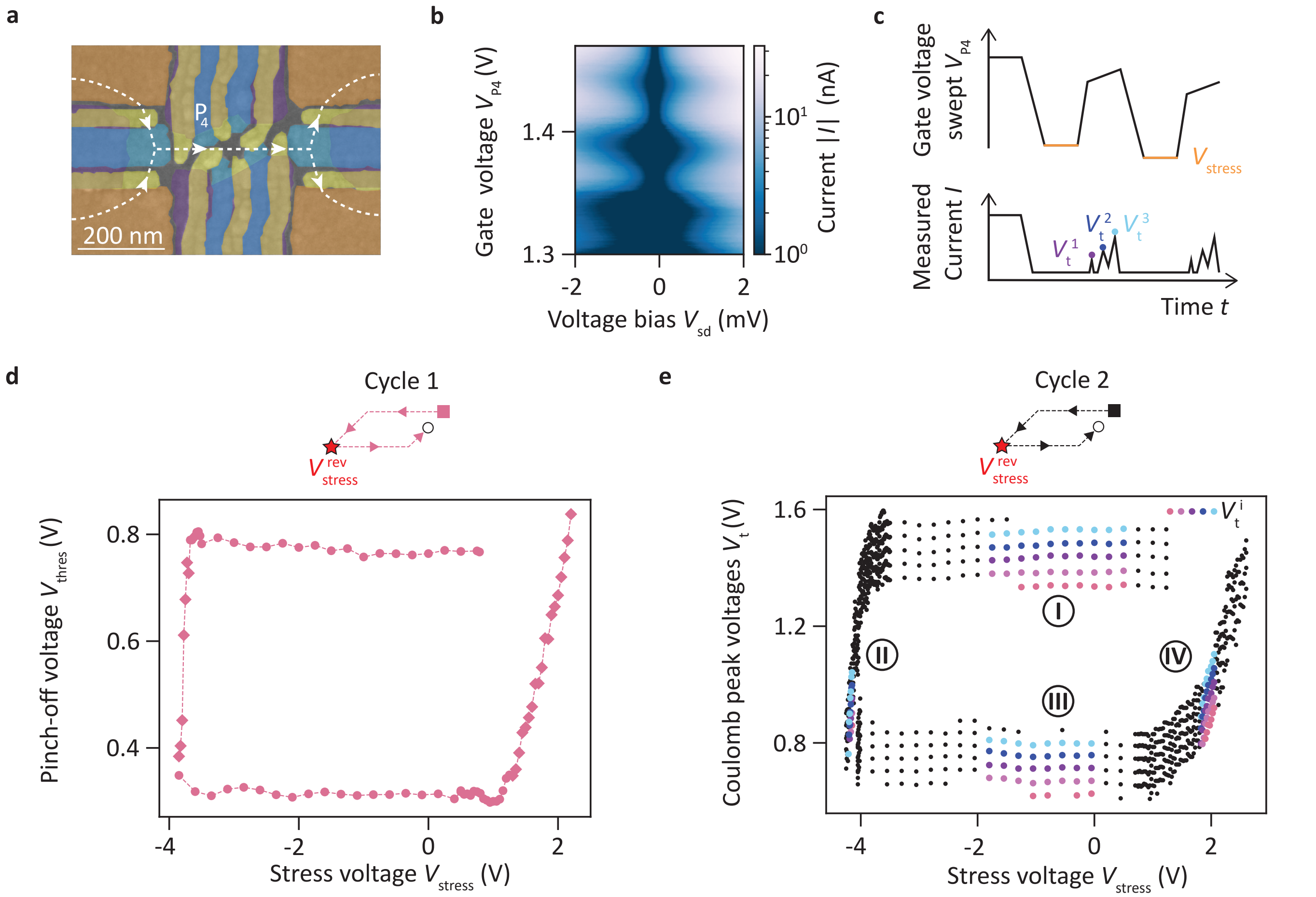

Figure S6. **Hysteresis of Coulomb-blockade oscillations.** **a**, Scanning electron micrograph of the device studied (device F). The plunger gates are colored in blue, the barrier gates in yellow, the accumulation gates in orange and the screening gates in violet. The plunger gate used during the experiment is labelled P_4. The current flow is depicted by the dashed line. **b**, Coulomb diamonds revealing a quantum dot formed under the plunger gate P_4. The colour scale is cut off below 1 nA. **c**, Schematics of the stress voltage sequence applied to gate P_4 and the current measured to obtain the data displayed in **e**. After the application of a stress voltage, the full pinch-off curve is taken and the first few Coulomb peaks are detected. **d**, V_{thres} hysteresis cycle measured before the quantum dot formation. The stress voltage cycle is depicted schematically on top. The square and the circle mark the starting point and the ending point of the cycles, respectively. The stress voltage $V_{\text{stress}}^{\text{rev}}$ upon which the stress voltage sequence is reversed is indicated by a star. Diamonds mark the data points from which the slopes $dV_{\text{thres}}/dV_{\text{stress}}$ of the cycle flanges are extracted. **e**, Hysteresis of Coulomb-blockade oscillations. Black dots show the first five detected peaks of the Coulomb-blockade oscillations as a function of the stress voltage V_{stress} applied to the gate P_4. Four regimes are marked with I, II, III, and IV. For each regime individual peak voltages are coloured to visualize the evolution of a specific charge transition voltage V_{t}^{i} with V_{stress}. The stress voltage cycle is depicted schematically on top. We note that we accidentally defined a sequence that after reaching the reversal point $V_{\text{stress}}^{\text{rev}} = -4250$ mV and before applying an increasing stress voltage sequence entailed another decreasing stress voltage sequence from -4010 mV to -4050 mV.

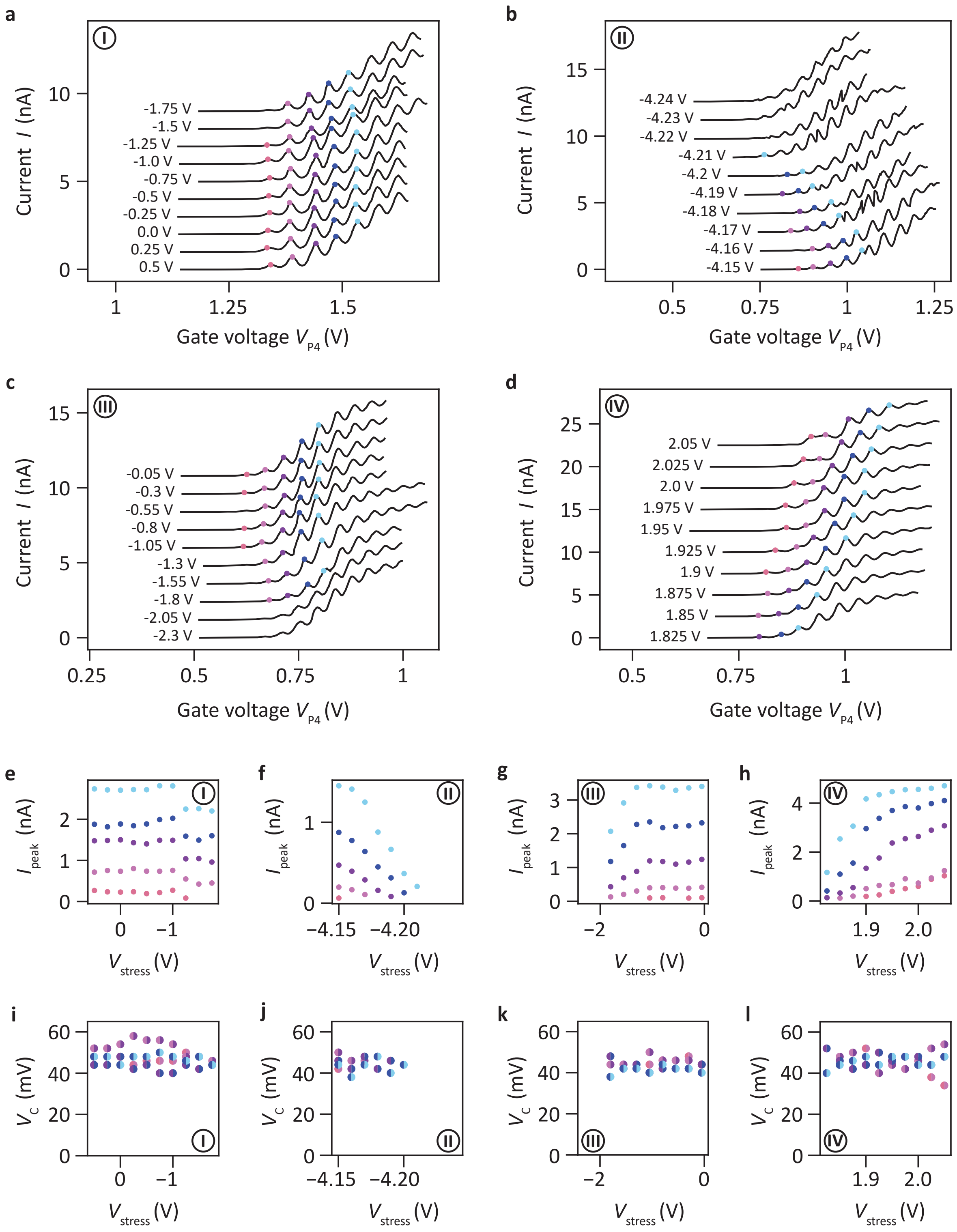

Figure S7. **Identification of individual transitions V_t^i. a-d**, Selected I/V-curves of the four regimes labeled in Fig. S6.e. The curves are offset to each other by 1.0, 1.4, 1.2 and 2.5 nA, respectively. The voltages given on the left side of the curves indicate the stress voltage V_{stress} applied just before the curve was recorded. Selected charge transitions V_t^i are identified and coloured to allow to follow their evolution with the applied voltage stress. In **a** and **c**, corresponding to parts I and III in Fig. S6.e, respectively, the peaks are labeled such that they show a minimal change in position. In **b** and **d**, corresponding to parts II and IV, respectively, the peaks are labeled such that their respective peak heights show a monotonous dependence with the applied stress voltages (decreasing in **b** and increasing in **d**). **e-h**, Corresponding peak heights I_{peak} of the coloured peaks in **a-d**. I_{peak} is defined as the current measured on the top of the peak. **i-l** Charging voltages $V_c = V_t^{i+1} - V_t^i$ extracted for the labeled charge transitions V_t^i in **a-d**. The two colors each marker is composed of correspond to the charge transition voltages used to calculate V_c.

VIII. SAMPLES USED FOR EACH MEASUREMENT AND REVERSAL POINTS

Table T1 and Table T2 provide an overview of the different samples underlying the figures in this work, their gate design, the gates that were swept, and the reversal points $V_{\mathrm{stress}}^{\mathrm{rev}}$ after which the stress voltage sequence was reversed if applicable. The gate designs and gate names mentioned in Table T1 can be found in supplementary Fig. S1 and supplementary Fig. S6.a. Supplementary Fig. S3 shows gate designs and gate names for the SHTs referred to in Table T2.

Figure label	Device measured	Type of device	Gate(s) swept	Cycle number	Reversal/end point(s) $V_{\mathrm{stress}}^{\mathrm{rev/end}}$ of the stress voltage sequence (in V)
Fig 1	A	Linear array	P_1, P_2, P_3, P_4	N.A.	N.A.
Fig 2.b	B	Linear array	S_1	1	-3.7 / 1.7
Fig 2.c	A	Linear array	P_1	1	-2.4 / 2.4
				2	-2.4 / 2.5
				3	-2.3 / 2.6
				4	-2.3 / 2.7
				5	-2.6 / 2.1
Fig 3 (violet, light pink, dark pink curve)	C	3 × 3 array	S	N.A.	N.A.
Fig 3 (blue curve)	D	3 × 3 array	S	N.A.	N.A.
Fig 4	A	Linear array	P_1, P_2, P_3, P_4	N.A.	N.A.
Fig S2	D	3 × 3	S	1	-3.4 / 1.7
				2	-3.4 / 1.75
				3	-3.4 / 1.75
				4	-3.35 / 1.75
				5	-3.35 / 1.7
				6	-3.35 / 1.75
				7	-3.35 / 1.8
				8	-3.35 / 1.75
				9	-3.4 / 1.75
				10	-3.4 / 1.8
Fig S5	A	Linear array	P_1, P_2, P_3, P_4	N.A.	N.A.
Fig S6.d (V_{thres} hysteresis)	F	2 × 2 array	P_4	1	-3.85 / N.A.
Fig S6.e and Fig S7 (V_{t} hysteresis)	F	2 × 2 array	P_4	1	-4.25 (-4.05) / N.A.

Table T1. **Summary table for the Si/SiGe devices**

Figure label	SHT measured	Type of device	Gates swept	Cycle number	Reversal/end point(s) $V_{\mathrm{stress}}^{\mathrm{rev/end}}$ of the stress voltage sequence (in V)
Fig S3.b	I	4×4 array	S, B_1, B_2 (simultaneously)	1	1.5 / -1.0
				2	1.492 / -1.0
				3	-1.6 / -1.0
Fig S3.c	II	4×4 array	S, B_1, B_2 (simultaneously)	1	1.714 / -1.0
				2	1.8 / -1.0
				3	1.8 / -1.0
Fig S3.d	III	4×4 array	S, B_1, B_2 (simultaneously)	1	2.8 / 0.05
				2	2.8 / 0.1
				3	1.7 / -0.25
				4	2.7 / 0.0

Table T2. **Summary table for Ge/SiGe devices**

REFERENCES

[1] B. Paquelet Wuetz, M. P. Losert, S. Koelling, L. E. A. Stehouwer, A.-M. J. Zwerver, S. G. J. Philips, M. T. Mądzik, X. Xue, G. Zheng, M. Lodari, S. V. Amitonov, N. Samkharadze, A. Sammak, L. M. K. Vandersypen, R. Rahman, S. N. Coppersmith, O. Moutanabbir, M. Friesen, and G. Scappucci, Atomic fluctuations lifting the energy degeneracy in si/sige quantum dots, Nature Communications **13**, 7730 (2022).

[2] W. I. L. Lawrie, H. G. J. Eenink, N. W. Hendrickx, J. M. Boter, L. Petit, S. V. Amitonov, M. Lodari, B. Paquelet Wuetz, C. Volk, S. G. J. Philips, G. Droulers, N. Kalhor, F. van Riggelen, D. Brousse, A. Sammak, L. M. K. Vandersypen, G. Scappucci, and M. Veldhorst, Quantum dot arrays in silicon and germanium, Applied Physics Letters **116**, 080501 (2020).

[3] F. Borsoi, N. W. Hendrickx, V. John, S. Motz, F. van Riggelen, A. Sammak, S. L. de Snoo, G. Scappucci, and M. Veldhorst, Shared control of a 16 semiconductor quantum dot crossbar array, arXiv , 2209.06609 (2022).

[4] A. Sammak, D. Sabbagh, N. W. Hendrickx, M. Lodari, B. Paquelet Wuetz, A. Tosato, Y. LaReine, M. Bollani, M. Virgilio, M. A. Schubert, P. Zaumseil, G. Capellini, M. Veldhorst, and G. Scappucci, Shallow and undoped germanium quantum wells: A playground for spin and hybrid quantum technology, Advanced Functional Materials **29**, 1807613 (2019).

[5] M. Lodari, N. W. Hendrickx, W. I. L. Lawrie, T.-K. Hsiao, L. M. K. Vandersypen, A. Sammak, M. Veldhorst, and G. Scappucci, Low percolation density and charge noise with holes in germanium, Materials for Quantum Technology **1**, 011002 (2021).

[6] N. W. Hendrickx, D. P. Franke, A. Sammak, M. Kouwenhoven, D. Sabbagh, L. Yeoh, R. Li, M. L. V. Tagliaferri, M. Virgilio, G. Capellini, G. Scappucci, and M. Veldhorst, Gate-controlled quantum dots and superconductivity in planar germanium, Nature Communication **9**, 2835 (2018).
